# The trials and tribulations of a robotic screening core

**DOI:** 10.1155/S1463924695000095

**Published:** 1995

**Authors:** John Babiak, Brian Lucotch, Anthony Russo, Linda Heydt, Sharon Williams, Ronald McCaully

**Affiliations:** Wyeth-Ayerst Research C.N. 8000 Princeton NJ 08543 USA

## Abstract

It is well recognized within the pharmaceutical industry that high throughput screening is a valuable and rapid tool to identify novel chemical compounds that may lead to tomorrow's drugs. High throughput screening involves testing as many chemical compounds as quickly as possible against a defined molecular or cellular ‘target’ (for example an enzyme) in the hope that interacting compounds may provide significant therapeutic benefits.

At Wyeth-Ayerst Research, a Robotics and Automation Research Core Group has been established which serves as the in-house resource for high throughput screening. The robotics group has three missions: (1) develop and perform high throughput screens for customers in all therapeutic departments in the company; (2) educate customers in issues related to screen design; and (3) help customers to bring automated workstations into their laboratories. The mission, therefore, requires the effective use of automation, as well as building a strong collaboration with customers.

The challenges that have been faced fall into two categories: technology limiting and customer relations. Technological challenges arise because it is necessary to develop and implement assays with very different formats and biochemical endpoints within extremely shortened time frames. The primary means to meet these challenges is with flexible robotics and flexible people. Challenges in the area of customer relations include setting realistic expectations, maintaining a sense of collaboration (and not merely service), educating investigators as to how to deal with the huge amount of data generated and seeking feedback. Effective and frequent communication, and an awareness of each individual's perspective, are essential to provide the most appropriate service.


Journal of Automatic Chemistry, Vol. 17, No. 2 (March–April 1995), pp. 55–58

### The trials and tribulations of a robotic screening core

**John Babiak, Brian Lucotch, Anthony Russo**
**Linda Heydt, Sharon Williams and**
**Ronald McCaully**
*Wyeth-Ayerst Research, C.N. 8000, Princeton, NJ 08543, USA*

*It is well recognized within the pharmaceutical industry that high throughput screening is a valuable and rapid tool to identify novel chemical compounds that may lead to tomorrow's drugs. High throughput screening involves testing as many chemical compounds as quickly as possible against a defined molecular or cellular 'target' (for example an enzyme) in the hope that interacting compounds may provide significant therapeutic benefits.*

*At Wyeth-Ayerst Research, a Robotics and Automation Research Core Group has been established which serves as the in-house resource for high throughput screening. The robotics group has three missions: (1) develop and perform high throughput screens for customers in all therapeutic departments in the company; (2) educate customers in issues related to screen design; and (3) help customers to bring automated workstations into their laboratories. The mission, therefore, requires the effective use of automation, as well as building a strong collaboration with customers.*

*The challenges that have been faced fall into two categories: technology limiting and customer relations. Technological challenges arise because it is necessary to develop and implement assays with very different formats and biochemical endpoints within extremely shortened time frames. The primary means to meet these challenges is with flexible robotics and flexible people. Challenges in the area of customer relations include setting realistic expectations, maintaining a sense of collaboration (and not merely service), educating investigators as to how to deal with the huge amount of data generated and seeking feedback. Effective and frequent communication, and an awareness of each individual's perspective, are essential to provide the most appropriate service.*

### Evolution of the perception of the robotics group

Papers at previous ISLARs have suggested that skillful management of laboratory automation is critical to the successful application of robotics in the pharmaceutical industry. The major management issues addressed in earlier presentations have reflected the novelty of robotics in the laboratory setting. Common topics have included:

(1) Senior management approval.

(2) The right technical champion.

(3) The right political champion.

(4) Alienation of laboratory personnel.

Presentations on these topics have stressed the hurdles one faces when introducing robotics into an industrial division and have provided suggestions for how to become successful. Recent ISLAR discussions on whether to centralize or decentralize robotics were among the first to present a perspective that assumed success and, rather, focused on broadening the scope of robotics in the laboratory.

The perception of laboratory robotics by pharmaceutical industry senior management has evolved over time, particularly regarding the application of robotics to high throughput drug screening. The impressive successes claimed by screening groups in both large and small pharmaceutical companies has loudly announced the high level of achievement that is feasible. The robotics group is no longer viewed as purely an exploratory, high-risk venture with a narrow focus. Instead, laboratory robotics is increasingly viewed by senior management as a low-risk investment that will deliver value to the organization as a new, all-purpose screening strategy for drug discovery. Thus, the robotic drug screening group must now play the role of a service facility that is expected to provide its scientist customers within the organization with a variety of products in a predictable, timely and cost-effective manner.

### Issues for the robotics group as service core

The Robotics and Automation Research Global Core Group was formed at Wyeth-Ayerst Research in January, 1994 and has three goals: to serve as a central high throughput screening facility for all therapeutic areas; to serve as a screen design resource—including new technologies—for high throughput screening; and to serve as a robotics/automation resource for discovery research.

High throughput screening is defined as testing as many chemical compounds as quickly as possible against a defined molecular or cellular 'target' (for example enzyme, cellular receptor, DNA transcription factor, cellular adhesion molecule) in the hope that interacting compounds may provide significant therapeutic benefit.

The mission to meet all three goals has two key elements: (1) making effective use of automation; and (2) building strong collaborations with scientist customers.

The robotics screening group within the pharmaceutical industry faces a new set of issues because of its new perceived role as a service facility, such as:

(a) Working with suppliers.

(b) Defining its products.

(c) Competition.

(d) Advertising.

(e) Customer service.

(f) Customer complaints.

This paper was presented at the 1994 ISLAR meeting.

© Zymark Corporation 1995

The concept of the internal customer has become a familiar paradigm within many industries to underscore the idea that the optimal performance of each individual's job provides value to others within the organization. Two factors complicate the emerging role of the robotic screening group as a service provider. One is simply that scientist customers have unique needs. The other is that the skills needed to create a successful robotics application are not the same required to provide services to customers. We have summarized these two factors as two irreverent mottoes: 'scientists make lousy customers' and 'scientists make really lousy service personnel'. Scientific training tends to emphasize characteristics of self-confidence, perseverance and the ambition to compete for grants, tenure, publications and recognition for new discoveries. These people are inclined neither to be patient while receiving mediocre service, nor be willing to deliver impeccable service to others of similar ilk.

The six issues for the robotic group service core noted above are not unique. The purpose of this paper is not merely to draw attention to situations and problems that are quite familiar to members of robotic screening groups throughout the pharmaceutical industry. More importantly, we hope to offer a new perspective that will suggest options for dealing with each issue based upon our limited experience to date.

### The three value disciplines for success

A good beginning is a thought-provoking article by Michael Treacy and Fred Wiersema [1]. The authors describe three distinct qualities called 'value disciplines' that exemplify the image a successful company might possess with its customers, namely:

(1) Operational excellence.

(2) Product leadership.

(3) Customer intimacy.

Operational excellence is convenience driven. A company that excels at operational excellence provides inexpensive and predictable products and services with little emphasis on customization. One example would be Wal Mart.

Product leadership is innovation driven. Customers of a company that is known for product leadership expect to pay more, but know that they will always receive creative, state-of-the-art products that will yet be replaced by newer products. An example would be the sports shoe manufacturer, Nike.

Customer intimacy is satisfaction driven. A company that emphasizes customer intimacy will provide detailed service to each customer regardless of the value of the specific purchase. Customers feel satisfied that every question has been answered and that they have purchased exactly what they need. Examples would be Nordstroms and Home Depot.

Clearly, operational excellence, product leadership and customer intimacy are all important qualities that customers appreciate. The premise of the article by Treacy and Wiersema is, however, that market leaders are usually those companies that excel in just one of the three value disciplines. Surely, these successful companies do not ignore the other value disciplines, but the market leaders do not try to be all things to all customers. Rather, being exceptional in a certain class of service (a value discipline) is the key to success.

In forming our robotic screening group, we made a conscious effort to adopt the value discipline of customer intimacy. We felt, at the time, that we lacked the experience and resources to make high throughput screening convenient for all customers. Also, although we try to use state-of-the-art equipment and software, our customers really want high quality data in a timely manner. As a new robotic screening group, we focused on customer intimacy because we wanted our customers to learn and work with us in our effort to become a group that delivers value to Pharmaceutical Discovery Research, one project at a time.

### Working with suppliers

A robotics screening group deals with many suppliers which are critical to the group's ability to deliver its screening services to its scientist customers. Typical needs that are provided by suppliers include: compound sourcing; hardware; software; robotic system integration; reagents and plastics; data management—local; and data management—corporate.

Many issues associated with these needs are important, but only a few are addressed in this paper.

A major issue faced by a robotic screening group is the need for a reliable source of compounds for screening. To address this issue the robotic screening group at Wyeth-Ayerst Research is under the same leadership as the Compound Room. Earlier this year the robotics group designed an automated system for the Compound Room that will automate compound weighing, dissolution, distribution into microplates, bar coding and inventory recording. By working together as partners, a long-term solution to the need for compounds is in progress.

Major needs, such as effective data management and reliable system integration, are especially critical. We have tried to perform much of this work within our own group, when possible, and are in the process of developing our own robotics LAN to ensure data integrity.

### Defining the products

As a new group within the company providing a new service, there was initially some confusion as to what our customers wanted and expected. Very early in our existence, we performed a customer survey to learn what types of screens the investigators thought might be useful for their research project. Quite expectedly, we learned that customers wanted us to be able to perform screens of all types of formats and endpoints. Our goal, therefore, was to bring the right capabilities on line first and educate each customer as to how we can help them during our period of growth and development.

The robotic screening group must answer the simple question: what can we deliver and when? Starting with limited resources, we started with assays that are easy to do and are gradually expanding our repertoire. We also insist on a commitment by customers to provide us with key reagents and, sometimes, personnel. In return, we make a concerted effort to provide the scientist customer with an honest appraisal of capabilities, time frames, resource needs, potential pitfalls and limitations.

As a first step toward working with each customer, we ask that a brief but detailed proposal form be completed, even before the scientist has begun working on the basic assay. The form asks leading questions regarding assay characteristics, expectations, urgency and level of commitment. Soon after receiving the proposal we arrange a follow-up group discussion (including all members of the robotic screening group) on the science, format, scheduling, statistics and philosophy of the proposed screen. We also provide the investigator with our written assay criteria guidelines which address details that are essential for efficient automation of many types of screens. The essence of the forms, discussions and guidelines is frequent communication between the robotics group and the potential customer.

One of the goals of the robotics group is to identify and explore new technologies that may make current screens more efficient or provide solutions for unmet screening needs. As a part of this effort, the robotics group has invited speakers to discuss biophysical endpoints and new hardware options. These presentations have been well received by customers and have encouraged them to think more broadly when considering new assays.

### Competition

There are two examples of competition to a robotic screening group, neither of which is actually a threat:

(1) Corporate alliances with specialized companies that perform screening.

(2) Automation in customers' laboratories.

Corporate screening alliances that benefit the parent company should always be encouraged. These external alliances increase customer awareness of the value of robotic screening and provide free advertising for the internal robotics group. Specialized companies frequently work in particular niches, so it is easy to avoid overlap. Lastly, external alliances may allow for technology transfer opportunities that can enhance the internal robotics capabilities.

Automation in customers' laboratories should be welcomed and encouraged. Members of our robotics group play a role in introducing automation into other labs and thereby establish the group's reputation as an internal consultant and increase customer awareness of the group's services. Ultimately, scientist customers who are familiar with automated equipment will know how to create screens that are readily robotizable. Thus, automation in investigators' labs could lead to more efficient development of screens run by the robotic screening group.

### Advertising

Since the robotics screening group is a service facility, advertising contributes to maintaining the steady stream of customers essential for success. The only detrimental advertising is top down. Customers who use the screening services because they were told to will never be satisfied customers. Avoid senior management mandates when possible. All other type of advertising are usually constructive and fun.

The means of advertising that we have employed include word of mouth, inviting guest speakers, working with scientists who want automation in their own labs, circulating unique biophysical endpoint articles or advertisements to investigators with special needs, providing many tours and hosting a robotics open house. Ultimately, there are only two forms of advertising that count: results and satisfied customers. Data quality is absolutely essential. Delivering no data is preferable to delivering bad data. And the customer who is pleased with the quality of the data you have produced will provide an enormous amount of free and beneficial advertising for the robotics group.

### Customer service—failures

Most customer service failures are the result of poor communication: unrealistic predictions, especially time frames; conflicting information from multiple sources; no information during critical periods.

All of these problems are preventable. When they occur, they must be dealt with immediately in a direct and personal manner. Some pointers:

(1) Explain everything to customers several times.

(2) Pay attention to networking and reputation.

(3) Document major agreements with customers.

(4) Communicate problems promptly.

(5) Be responsive to questions and concerns.

The other major customer service failure is delivering bad data. Any extra resources expended to ensure quality assay performance and maintain data integrity are well worth the cost.

### Customer service—successes

Customer service successes are easy to identify and are somewhat self-explanatory:

(1) Participatory customers.

(2) Designate primary contacts on each side.

(3) Cautious predictions.

(4) Honesty (even for bad news).

(5) Tours.

(6) Invited speakers.

(7) Exploratory research.

(8) Good quality data.

### Summary and advice

After not quite one year of operation we have recorded both successes and failures. Although we still have far to go, we have already run and completed screens and developed a reputation for providing new ideas regarding automation and screen design. Our progress has been the result of technical expertise, hard work and a conscious effort to excel in customer intimacy. To us, this means: frequent communication in many oral and written forms; demonstrating enthusiasm for each new project; educating customers in automation, screening formats and data management; and promoting realistic expectations.

In summary, we would like to provide three pieces of advice which have contributed to our progress as a service facility for which the customer is always right: define both your technical and service goals; understand and advertise your strengths and your weaknesses; and educate your customers so that she or he *will* be always right!

### References

1. Treacy, M. and Wiersema, F., Customer intimacy. *Harvard Business Review* (1993).

